# Utilisation of rehabilitation due to mental disorders during the SARS-CoV-2 pandemic: a difference-in-differences analysis

**DOI:** 10.1186/s12888-023-04627-w

**Published:** 2023-03-06

**Authors:** Matthias Bethge, David Fauser, Pia Zollmann, Marco Streibelt

**Affiliations:** 1 Institute for Social Medicine and Epidemiology, University of Lübeck, Ratzeburger Allee 160, 23562 Lübeck, Germany; 2 Federal German Pension Insurance, Berlin, Germany

**Keywords:** Rehabilitation, Mental health services, Mental disorders, SARS-CoV-2

## Abstract

**Background:**

Our analyses examined the extent to which the use of rehabilitation for patients with mental disorders decreased due to the COVID-19 pandemic in Germany.

**Methods:**

We used monthly cross-sectional administrative data on rehabilitation utilisation due to mental disorders in 2019 and 2020 and estimated a difference-in-differences model to determine the reduction in rehabilitation utilisation attributable to the pandemic.

**Results:**

We included 151,775 rehabilitations in 2019 and 123,229 rehabilitations in 2020 in our analysis. The number of rehabilitations decreased from April to December by 14.2% due to the pandemic (March to December: 21.8%). The decline was more pronounced for women than for men and varied regionally. Temporal and regional differences in utilisation were moderately associated with the decrease in mobility in the pandemic year. In the first phase of the pandemic, i.e., March and April 2020, the decline was strongly associated with the regional incidence of SARS-CoV-2 infection.

**Conclusion:**

Due to the pandemic, significantly fewer rehabilitations due to mental disorders occurred in Germany in 2020 than in 2019. The likely increasing need for rehabilitation for people with mental disorders should be addressed by making rehabilitation access and delivery more flexible.

## Introduction

The SARS-CoV-2 pandemic has posed major challenges to the care of people with mental disorders. Psychosocial and psychiatric care was reduced substantially around the world. Treatments were abruptly terminated, interrupted, or replaced by telemedicine care [1–3]. Elective admissions to psychiatric hospitals have largely ceased [4]. At the same time, contact restrictions and concomitant feelings of loneliness, as well as insecurity and the loss of routines, often led to a deterioration in the mental state of people with mental disorders [5].

Unemployment and underemployment due to the pandemic, as well as financial worries, can also have a psychological impact [6]. Existing social inequalities that disadvantage people with mental disorders are therefore likely to increase. A meta-analysis of 48 studies recently estimated that the prevalence of major depression and anxiety disorders increased by more than 25% during the pandemic, and that cases of major depressive disorders increased by 53 million and anxiety disorders by 76 million worldwide [7].

Considerable restrictions on the care of people with mental disorders were observed in Germany. About half of the people with a current depressive episode reported constraints in medical care, including care by specialists and psychotherapists, during the first year of the pandemic [8]. The inpatient treatment capacity of psychiatric clinics was temporarily reduced to 60% of the pre-pandemic capacity. The day clinic and outpatient services offered by psychiatric clinics were likewise only available to a limited extent or were discontinued completely [9]. Similar findings were reported from regional hospital networks [10].

In addition to restrictions on primary care and outpatient and inpatient psychiatric treatment, potential effects on rehabilitation care and aftercare are relevant for people with mental disorders [11, 12]. Rehabilitation is mentioned in the German guidelines for the treatment of unipolar depression and anxiety disorders and is aimed in particular at maintaining the quality of life as well as securing and restoring work ability and returning to work [13]. The sudden changes in working life caused by the pandemic (including short-term working, home working, and digitalisation) are likely to lead to further barriers to inclusion, especially for many people with mental disorders [4]. Multimodal rehabilitation is particularly appropriate for addressing these problems.

The admission bans in many rehabilitation facilities introduced in spring 2020 had an immediate and direct impact on the utilisation of rehabilitation in Germany. However, the decline in utilisation of rehabilitation for mental disorders due to the pandemic has not been comprehensively described to date. Describing the decline is important for estimating unmet need during the pandemic and any additional treatment capacity that may be needed in the near future.

We therefore compared the number of rehabilitations due to mental disorders performed in Germany in 2020 with the number performed in 2019, in order to clarify the extent to which utilisation of rehabilitations declined after the German Bundestag declared an epidemic situation of national concern on March 27, 2020. In addition, we analysed the differences in the decline in rehabilitation utilisation by gender and region. Furthermore, we examined possible correlations of the decline in rehabilitation utilisation with the observed mobility decline, the incidence of SARS-CoV-2 infections in Germany, and socioeconomic indicators.

## Methods

### Study design

We used monthly cross-sectional data on rehabilitation of working aged people due to mental disorders performed in 2019 and 2020 for a difference-in-differences analysis [14, 15]. Preparation of the manuscript followed the Strengthening the Reporting of Observational Studies in Epidemiology (STROBE) Reporting Guideline [16]. Since only aggregated data were used, the study was not reviewed by an ethics committee.

### Setting

In Germany, rehabilitation of working aged people due to mental disorders is mainly provided by the pension insurance institutions. Rehabilitation is not prescribed by physicians or psychotherapists, but instead involves a request from the individual. Utilisation, therefore, depends on whether people with a need for rehabilitation decide in favour of or against an application [12, 17]. The number of rehabilitations carried out by pension insurance institutions due to mental disorders has roughly doubled in Germany over the past 20 years [18]. In 2019 and 2020, 16% and 17% of the rehabilitations performed were due to mental disorders. The most common disorders are affective disorders and adjustment disorders [19]. Most rehabilitations due to mental disorders are carried out as inpatient programmes and last five weeks.

### Data

Complete data on the use of rehabilitation due to mental disorders were provided by the German Pension Insurance, categorised by gender, state, and month of use, including rehabilitations starting and ending within 2019 and 2020. Regional population data were retrieved from the statistical information system of the Federal Statistical Office. We obtained the incidence of SARS-CoV-2 infections from the Robert Koch Institute reporting system. Mobility was captured through anonymised and aggregated mobile phone data. These data are also provided by the Federal Statistical Office, mapping the movements within a region based on switching between radio cells. Regional socioeconomic indicators (i.e. unemployment rates and net household income) were used from the statistical information system of the Federal Institute for Research on Building, Urban Affairs and Spatial Development.

### Statistical analysis

The rehabilitations provided were first presented descriptively by gender, state and month. Then, using a difference-in-differences model, we estimated the reduction in rehabilitation utilisation attributable to the pandemic [15, 20]. The difference-in-differences approach uses repeated cross-sectional data collected before and after an event (e.g., law reform or pandemic outbreak) [14, 15]. The difference-in-differences estimator corresponds to the difference of two before-after changes observed in an exposed and a non-exposed group. While usually a control group in the same time frame is used, this approach is also appropriate if an earlier time frame is employed as control group [21, 22]. As the rapid spread of the pandemic did not allow for unexposed comparison regions, the impact of the pandemic could only be estimated by comparison with an earlier time frame. In our analyses, the exposed group was therefore represented by January through December of the 2020 pandemic year, and the unexposed comparison group was represented by January through December 2019. The first quarter of each year, January through March, was categorised as the pre-observation period. The months of April through December were the post-observation period. The distinction between the pre- and post-observation periods was due to the fact that the German Bundestag declared the epidemic to be of national concern on March 27, 2020, and we expected a decrease in rehabilitations following this declaration. The difference-in-differences estimator was determined as the interaction term of observation period and year using a Poisson regression model. The incidence rate ratio (IRR) was calculated as the difference-in-differences estimator. The IRR relates the ratio of the mean rehabilitation utilisation between the post- and pre-observation periods of 2020 to the ratio of the mean rehabilitation utilisation between the post- and pre-observation periods of 2019. The value of 1 - IRR describes the proportion by which the number of rehabilitations due to mental disorders in 2020 decreased on average in the post-observation period due to the pandemic.

In addition to the difference-in-differences estimator for the total population, we also determined difference-in-differences estimators for men and women and for the different federal states. Regional differences in the decline in rehabilitations were presented using choropleth maps. For this purpose, the decline was categorised along quartiles. To test the association of decline in mobility and incidence of SARS-CoV-2 infections with rehabilitation utilisation decline, we calculated Spearman’s rho. To test associations of socioeconomic indicators with rehabilitation utilisation decline, we also calculated Spearman’s rho. We supplemented our analysis with a sensitivity analysis, for which we used a pre-observation period from January to February and a post-observation period from March to December. The two-sided probability of error was 5%. All analyses were performed using Stata/SE version 16.0.

## Results

### Sample

From January to December 2020, 126,512 rehabilitations were used due to mental disorders. In 2019, there were 154,427 rehabilitations. We excluded a total of 5,935 rehabilitations due to incomplete information on place of residence, place of residence abroad, or undetermined gender. Of the remaining 123,229 rehabilitations in 2020, 77,302 (62.7%) were for women. Of the remaining 151,775 rehabilitations in 2019, 96,127 (63.3%) were used by women.

### Reduction of use of rehabilitation

**Fig. 1:**
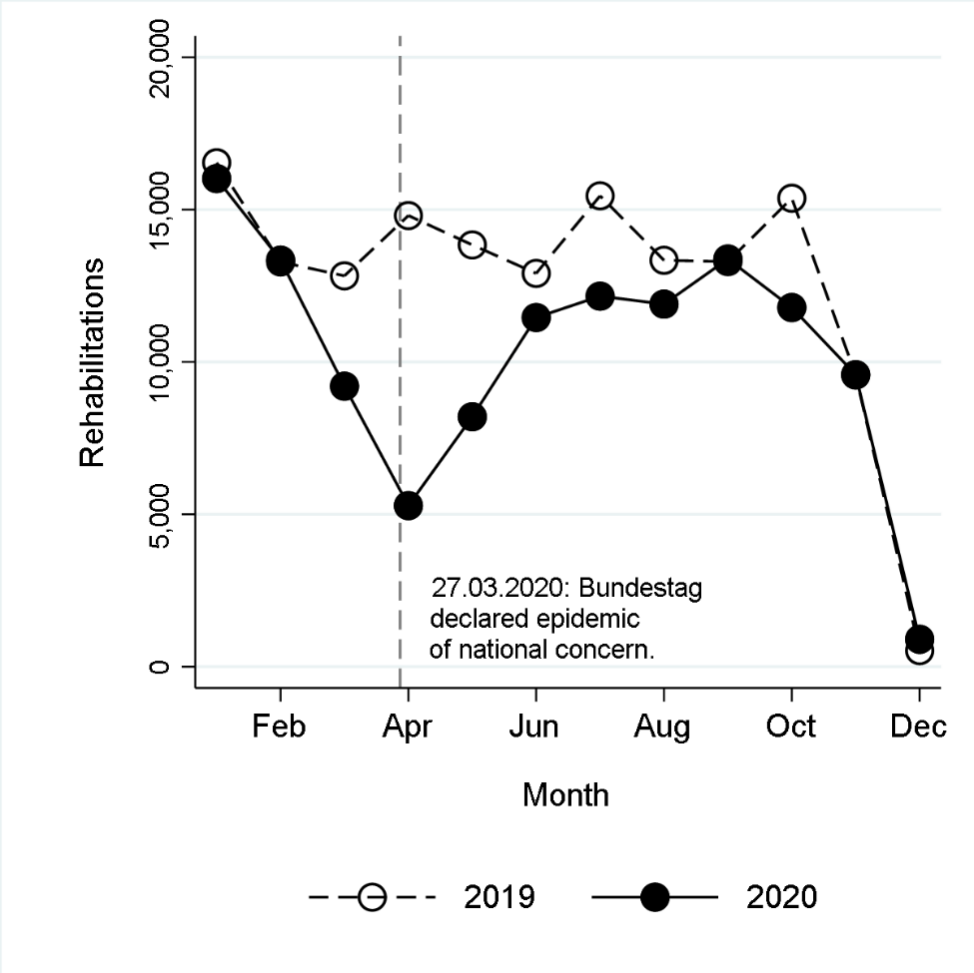
Number of rehabilitations due to mental disorders in 2019 and 2020

**Fig. 2:**
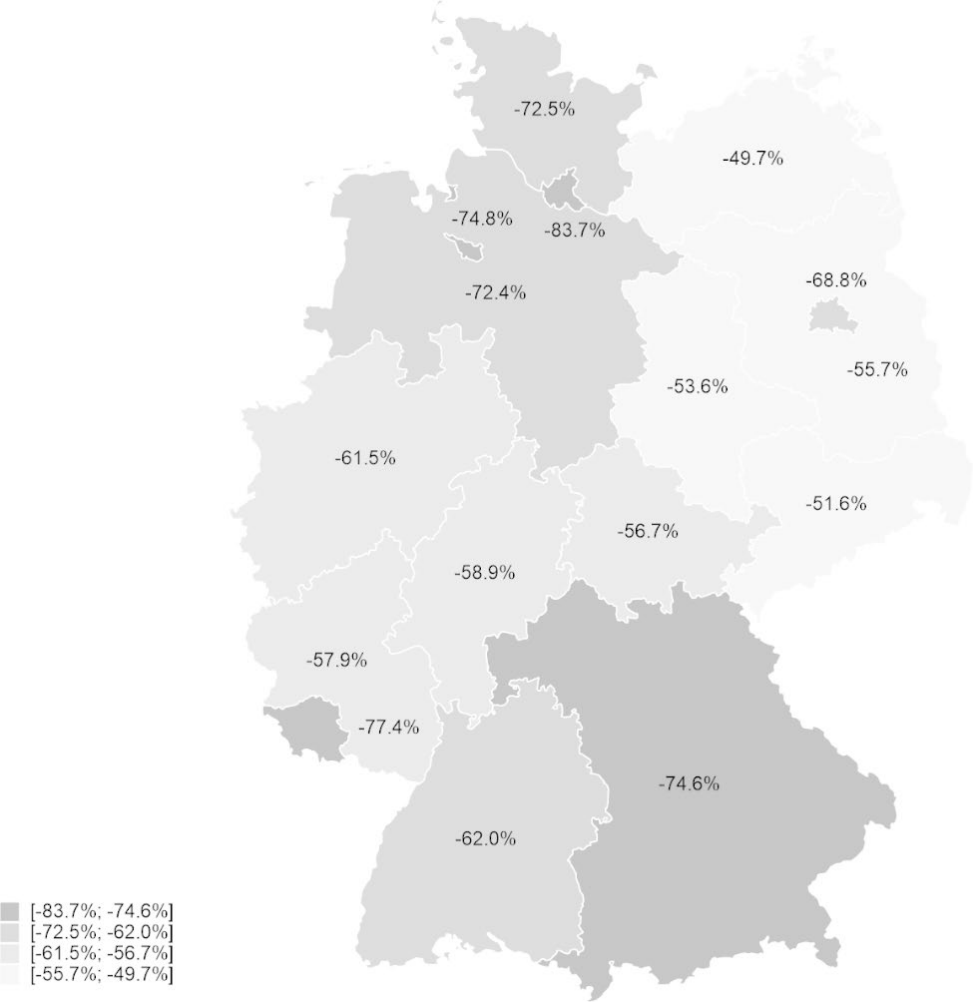
Decline in rehabilitations performed in April 2020 compared to April 2019 by federal state

Compared with 2019, the nationwide decline in rehabilitations performed in the pandemic year 2020 peaked at 64.3% in April (Fig. 1; Table 1). The decline in April was higher for women (68.0%) than for men (57.9%). By federal state, the decline varied from 49.7% in Mecklenburg-Western Pomerania to 83.7% in Hamburg (Fig. 2).

**Table 1:** Number of rehabilitations due to mental disorders and change between 2019 and 2020

	Number of rehabilitations		Change	
	2019	2020	absolute	relative
**January**	16,542	16,021	-521	-3.1%
**February**	13,283	13,343	60	0.5%
**March**	12,829	9,206	-3,623	-28.2%
**April**	14,807	5,286	-9,521	-64.3%
**May**	13,843	8,203	-5,640	-40.7%
**June**	12,908	11,459	-1,449	-11.2%
**July**	15,455	12,161	-3,294	-21.3%
**August**	13,338	11,900	-1,438	-10.8%
**September**	13,294	13,381	87	0.7%
**October**	15,376	11,787	-3,589	-23.3%
**November**	9,573	9,584	11	0.1%
**December**	527	898	371	70.4%
**Total**	151,775	123,229	-28,546	-18.8%

Following the sharp decline in rehabilitations provided in April 2020, rehabilitation utilisation had risen again by September. In September 2020, the previous year’s level was reached. In October 2020, utilisation was again well below the previous year’s level, while in November—as previously in September—utilisation was in line with 2019. In December, the number of rehabilitations provided was low in both years (Fig. 1; Table 1).

### Difference-in-differences estimators

Nationwide, rehabilitation utilisation decreased by 14.2% (IRR = 0.858; 95% CI: 0.844 to 0.872) for April to December (Table 2). The decrease was significantly greater for women, at 15.9% (IRR = 0.841; 95% CI: 0.823 to 0.858), than for men, for whom a decrease of 11.2% (IRR = 0.888; 95% CI: 0.865 to 0.913) was observed. At 28.4% and 23.4%, the decline was most pronounced in Bremen and Mecklenburg-Western Pomerania. In Berlin and Brandenburg, no decrease in the number of rehabilitations was observed (Table 2). Our sensitivity analyses determined a reduction in rehabilitations for March to December of 21.8% (IRR = 0.782; 95% CI: 0.768 to 0.796).

**Table 2:** Difference-in-differences estimator for the decrease in rehabilitations due to mental disorders

	Primary analysis		Sensitivity analysis	
Population	IRR	95% CI	IRR	95% CI
**Total**	0.858	0.844, 0.872	0.782	0.768, 0.796
**Gender**				
**Women**	0.841	0.823, 0.858	0.762	0.745, 0.780
**Men**	0.888	0.865, 0.913	0.816	0.792, 0.841
**Federal state**				
**Baden-Wuerttemberg**	0.886	0.847, 0.926	0.815	0.775, 0.856
**Bavaria**	0.851	0.809, 0.895	0.781	0.739, 0.826
**Berlin**	1.059	0.972, 1.153	0.947	0.862, 1.041
**Brandenburg**	1.005	0.920, 1.097	0.950	0.862, 1.048
**Bremen**	0.716	0.601, 0.852	0.674	0.555, 0.817
**Hamburg**	0.795	0.702, 0.899	0.733	0.639, 0.840
**Hesse**	0.803	0.756, 0.853	0.730	0.682, 0.781
**Mecklenburg-Western Pomerania**	0.766	0.683, 0.859	0.698	0.615, 0.793
**Lower Saxony**	0.821	0.783, 0.861	0.760	0.721, 0.800
**North Rhine-Westphalia**	0.854	0.827, 0.883	0.762	0.735, 0.789
**Rhineland-Palatinate**	0.897	0.835, 0.963	0.804	0.742, 0.870
**Saarland**	0.810	0.707, 0.928	0.717	0.618, 0.832
**Saxony**	0.856	0.786, 0.931	0.771	0.702, 0.846
**Saxony-Anhalt**	0.829	0.742, 0.926	0.753	0.665, 0.852
**Schleswig-Holstein**	0.865	0.792, 0.943	0.797	0.723, 0.877
**Thuringia**	0.813	0.729, 0.907	0.807	0.715, 0.911

IRR, incidence rate ratio; CI, confidence interval

### Regional mobility and incidence of SARS-CoV-2 infection

The monthly regional decline in rehabilitations due to mental disorders was moderately associated with the monthly regional decline in mobility (Spearman’s rho = 0.343, p < 0.001). The monthly regional incidence of SARS-CoV-2 infections was associated with the decline in rehabilitations only in March and April (March: Spearman’s rho = -0.626, p = 0.009; April: Spearman’s rho = -0.706; p = 0.002).

### Socioeconomic indicators

Regional unemployment rates in 2019 were not associated with the monthly decline in rehabilitations. However, a higher regional net household income in 2019 was associated with a stronger decline in rehabilitations in April 2020 (Spearman’s rho = -0,638, p = 0,008).

## Discussion

Our difference-in-differences analysis showed that the number of rehabilitations due to mental disorders decreased by 14.2% due to the pandemic for April to December. Our sensitivity analysis for March to December estimated a drop of 21.8%. The decline in utilisation was more pronounced for women. Temporal and regional differences in utilisation were moderately associated with the mobility decline in the pandemic year. We observed a strong association with regional incidence of SARS-CoV-2 infection in March and April.

The decline in rehabilitations for patients with mental disorders that we describe, particularly in the spring of 2020, is consistent with the decline in inpatient and outpatient care observed in the United States [23, 24], France [25] and the United Kingdom [26]. The reduced use of rehabilitation and other services for people with mental disorders is thus in striking contrast to the increase in mental illness observed during the pandemic [7]. The causes of the observed decline are manifold. In Germany, the substantial decline from March 2020 onward was mainly due to the temporary admission ban recommended by the pension insurance providers in the second half of March and maintained until mid-May. In the following months, the possibilities for admitting rehabilitation patients were significantly restricted due the mitigation strategies of the rehabilitation facilities.

In addition, the general restrictions that have been gradually introduced in Germany to control the incidence of infections and individual concerns about infections are likely to have had an effect on postponing the visits to a physician that are required for a rehabilitation application. This is supported by the observation that a decline of a similar extent was noted for other care services for people with mental disorders [27]. These analyses in the setting of other services have likewise described associations with reduced mobility. Similarly, the difference between women and men that we observed has been previously noted in other studies. These studies have described more pronounced protective behaviour among women [28–31]. In an eight-country study of nearly 22,000 participants, women were more likely to perceive the pandemic as a very serious health hazard, more likely to agree to restrictive measures, and more likely to comply with mitigation measures than men. A recent review found that these results were replicated in many studies [32]. Moreover, women bore the brunt of increased caregiving and domestic work during the pandemic. As known from other pandemics this also contributes to an increased risk of reduced healthcare access [33, 34].

The regional differences in rehabilitation decline, which we observed, were associated with mobility decline and the SARS-CoV-2 incidence. While we did not found associations with unemployment rates, our analyses demonstrated that a higher regional net household income was associated with a stronger decline in rehabilitations in April 2020. This finding reflects that the entry and spread of the virus in Germany began in the more prosperous western federal states of Germany (e.g. Bavaria and Hamburg). These states therefore responded earlier with constraints on public life explaining the initially greater decline in rehabilitations in these federal states.

The results of the current analysis must be interpreted considering the following limitations. First, our analyses only describe the extent of the reduction. They do not enable us to explain why people decided to refrain from using rehabilitation. Temporal and regional differences in the decline in mobility and spread of infection could only partially explain the variance in the reduction in rehabilitation use. Second, the period we included in the analyses is limited to the first wave of the pandemic, the summer with very low infection rates, and the first part of the second wave. The extent to which the described decline continued during the second wave, over the turn of 2020/2021, and in 2021 can only be clarified with future data. Third, administrative data are not generated primarily for research purposes and are responsive to changes in administrative processes, for instance how an incident case is determined [35]. Fourth, limiting rehabilitations in both years to rehabilitations completed in the same year underestimates the number of rehabilitations started in December in both years. This is likely to have diluted the true difference between the two years for this month.

These limitations are balanced by the following strengths. First, we were able to use validated administrative and publicly available data on rehabilitation utilisation, incidence and mobility, and socioeconomics for our analyses. Underreporting, overreporting and misreporting of rehabilitation utilisation are unlikely because of the use of administrative data, except for the data used for December. Second, the difference-in-differences approach we chose allowed us to investigate declines due to the pandemic.

For many people with a mental disorder in need of rehabilitation, not using rehabilitation increases the risk that their condition will persist or worsen. After the significant decline in rehabilitations in the spring of 2020, the number of rehabilitations increased again, but not to the extent that would be expected given unchanged disease burden or additional disease burden due to the pandemic. A rebound effect has not been observed so far. In view of the limited treatment capacities in rehabilitation centres for people with disorders, the increasing demand for people in need of rehabilitation due to postponement and abandonment, as well as new cases, is likely to lead initially to longer waiting times. Increasing the flexibility of treatment provision by making the duration of rehabilitation more flexible and expanding the digital treatment options developed and implemented during the pandemic could help to meet the expected increase in demand for rehabilitation for people with mental disorders. Future research should examine the gendered impact of the pandemic on access to health care and the long-term outcomes of unmet mental health care needs during the pandemic.

## Data Availability

The datasets used and/or analysed during the current study are available from the corresponding author on reasonable request.
